# Two-year follow-up of a clustered randomised controlled trial of a multicomponent general practice intervention for people at risk of poor health outcomes

**DOI:** 10.1186/s12913-024-10799-2

**Published:** 2024-04-19

**Authors:** Richard L. Reed, Leigh Roeger, Billingsley Kaambwa

**Affiliations:** College of Medicine and Public Health, Flinders University, GPO Box 2100, Adelaide, SA 5001 Australia

**Keywords:** Primary care, General practice, Economic evaluation, Health service use

## Abstract

**Background:**

This study was a two-year follow-up evaluation of health service use and the cost-effectiveness of a multicomponent general practice intervention targeted at people at high risk of poor health outcomes.

**Methods:**

A two-year follow-up study of a clustered randomised controlled trial was conducted in South Australia during 2018–19, recruiting 1044 patients from three cohorts: children; adults (aged 18–64 years with two or more chronic diseases); and older adults (aged ≥ 65 years). Intervention group practices (*n* = 10) provided a multicomponent general practice intervention for 12 months. The intervention comprised patient enrolment to a preferred general practitioner (GP), access to longer GP appointments and timely general practice follow-up after episodes of hospital care. Health service outcomes included hospital use, specialist services and pharmaceuticals. The economic evaluation was based on quality-adjusted life years (QALYs) calculated from EuroQoL 5 dimensions, 5 level utility scores and used an A$50,000 per QALY gained threshold for determining cost-effectiveness.

**Results:**

Over the two years, there were no statistically significant intervention effects for health service use. In the total sample, the mean total cost per patient was greater for the intervention than control group, but the number of QALYs gained in the intervention group was higher. The estimated incremental cost-effectiveness ratio (ICER) was A$18,211 per QALY gained, which is lower than the A$50,000 per QALY gained threshold used in Australia. However, the intervention's cost-effectiveness was shown to differ by cohort. For the adult cohort, the intervention was associated with higher costs and lower QALYs gained (vs the total cohort) and was not cost-effective. For the older adults cohort, the intervention was associated with lower costs (A$540 per patient), due primarily to lower hospital costs, and was more effective than usual care.

**Conclusions:**

The positive cost-effectiveness results from the 24-month follow-up warrant replication in a study appropriately powered for outcomes such as hospital use, with an intervention period of at least two years, and targeted to older people at high risk of poor health outcomes.

**Supplementary Information:**

The online version contains supplementary material available at 10.1186/s12913-024-10799-2.

## Introduction

An efficient and adequately resourced primary healthcare sector is recognised as critical for improved population health outcomes and for health funding to be sustainable [1]. In common with other developed nations, Australia has an ageing population, rising chronic and complex disease rates and a growing demand for expensive healthcare services [2]. These challenges are not unique to Australia and have encouraged local and international policy makers to consider wide-ranging health system reform [3].

Internationally, there is evidence that continuity of primary healthcare [4–6], longer general practitioner (GP) consultations [7] and timely general practice follow-up after episodes of hospital care [8, 9] are associated with better patient outcomes and health service utilisation. However, the evidence is mixed [10–12] and drawn mainly from observational studies. In addition, it is not clear whether initiatives to promote these elements would be beneficial in the Australian context.

In 2017, Flinders University was awarded a grant from The Royal Australian College of General Practitioners (RACGP) in collaboration with the Australian Government to conduct a clustered randomised trial to test whether a multicomponent general practice intervention for people identified by their GPs as being at high risk of poor health outcomes cost-effectively improved health outcomes and reduced the use of health services. The study enrolled patients with a preferred GP, longer GP appointments and timely general practice follow-up after major health events.

Flinders QUEST (QUality Enhanced general practice Services Trial) was successfully implemented during 2018–19, with 20 general practices and 92 GPs taking part [13]. Practices were cluster randomised on a 1:1 schedule to either the intervention or control (usual care) arm. Intervention group practices received a payment of A$1000 per enrolled patient to provide the intervention for a 12-month period. In all, 1044 patients were recruited from three cohorts: children and young people aged < 18 years (*n* = 58); adults aged between 18 and 64 years with two or more chronic diseases (*n* = 315); and older adults aged ≥ 65 years (*n* = 671). Further details to the methodological background to Flinders QUEST are provided in Additional file 1.

The outcomes, assessed after the 12-month intervention period, have been reported elsewhere [13]. Briefly, the intervention was not found to improve self-rated health (the primary outcome, measured using the visual analogue scale [VAS] of the EuroQoL 5 dimensions, 5 levels [EQ-5D-5L] questionnaire) or reduce health service use. The economic evaluation found that for the total sample the intervention was more effective in terms of quality-adjusted life years (QALYs; calculated from EQ-5D-5L utility scores) but was not cost-effective based on a willingness-to-pay (WTP) threshold of A$50,000 per QALY [14] gained. The intervention was cost-effective in a prespecified subgroup analysis of older people (**≥ **65 years) due primarily to lower hospital costs [13].

The design of Flinders QUEST incorporated a planned two-year follow-up to assess longer-term outcomes. The two-year follow-up study had two key aims. First, we wanted to determine whether an intervention effect for health service use (hospital service use, specialist services and pharmaceuticals) and cost-effectiveness may have emerged with a longer follow-up period. Second, we examined whether the cost-effectiveness of the intervention (QALY gains) observed at 12 months for older adults was sustained through to two years.

## Methods

### Description of the intervention

The multicomponent general practice intervention comprised enrolling patients with a preferred GP, providing patients access to longer GP appointments and timely (within 7 days) general practice follow-up after major health events. It also involved same-day appointments, where possible, for children and young people.

The operationalisation of the intervention in practices has been described in detail elsewhere [13] but is briefly outlined here. Trial patients were flagged in practice electronic software systems and prioritised to receive appointments with their preferred GPs and were to be offered a longer appointment. Appointments with the preferred GP were facilitated by practices reserving appointment slots specifically for trial patients. Practice nurses (PNs) or administrative staff checked each day for any hospital discharge summaries received (by fax) against their trial patient list and arranged follow-up appointments for patients discharged from hospital if this was clinically warranted.

During the study, a research PN helped practices with participant recruitment and implementation of the intervention. Intervention and control group practices received A$10,000 each to cover the administrative costs of patient recruitment, data collection and investigator meetings. Intervention practices also received a payment of A$1000 per enrolled patient to deliver the intervention over the 12-month intervention period. The payment of $A1000 per patient was designed to cover the costs that practices would incur implementing the intervention to a high standard including reserving appointments for trial patients to facilitate continuity of care, routinely offering long length appointments, and proactively following up patients after a hospital care episode. Qualitative interviews conducted with practice staff at the end of the intervention period found that practice staff believed that the intervention would not be possible to sustain financially when the trial payments ceased [15] and from this it can be inferred that without the payments very few (if any) practices would have agreed to take part in the study.

### Clinical effectiveness outcomes and statistical approach

The primary outcome of Flinders QUEST was the difference between the control and intervention groups in the change in self-rated health, measured using the VAS of the EQ-5D-5L questionnaire [16], between baseline and the 12-month follow-up. Self-rated health using the VAS was not assessed at the 24-month follow-up.

Secondary outcomes included hospital service use (the number of emergency department presentations, hospital admissions and hospital night stays), the number of specialist services and pharmaceuticals, as well as the intervention's cost-effectiveness. Data for the health service use outcomes were collected initially over two years: at baseline (the 12 months before the intervention) and at the 12-month follow-up. Ethics approval, based on individual-level informed patient consent, enabled further data extractions from SA Health public hospital records and from Services Australia for medical services and pharmaceuticals covering the 12 months after the intervention. The outcome measures collected across the three time periods are summarised in Table 1.

**Table 1:** Outcome and process measures collected at baseline and 12 and 24 months

	**Baseline**	**12 months**	**24 months**
**Primary outcome**			
Self-rated health (VAS)^a^	✓	✓	✗
**Secondary outcomes**			
Hospital service use			
ED presentations	✓	✓	✓
Hospital admissions	✓	✓	✓
Hospital night stays	✓	✓	✓
Specialist services	✓	✓	✓
Pharmaceuticals	✓	✓	✓
Cost-effectiveness analysis	✓	✓	✓
EQ-5D-5L	✓	✓	✗

^a^Data on self-rated health, determined using the visual analogue scale (VAS), were collected at the time of patient recruitment and at the 12-month follow-up. Baseline, the 12-month period prior to the intervention; 12 months, the 12-month intervention period; 24 months, the 12-month period following the intervention period; ED, emergency department; EQ-5D-5L, EuroQoL 5 dimensions, 5 levels questionnaire

Hospital service use, specialist services and pharmaceuticals were analysed using multilevel non-linear regression with random intercepts for practice and participant. The models estimated included group (categorised as intervention or control), time (categorised as baseline [the 12-month period prior to the intervention] and 24 months [the two-year period beginning from the start of the intervention]) and an intervention × time interaction.

The statistical analyses conformed to the intention-to-treat principle and were performed with two-sided *p* < 0.05 defined as statistically significant. Prespecified subgroup analyses were performed at the cohort (children, adults and older adults) level. No adjustment was made for multiple statistical testing. Analyses were performed using Stata 17.0. For further details relating to data sources and statistical methods for hospital service use, specialist services and pharmaceuticals see Additional file 2.

### Economic evaluation

#### Methods

Best practice guidelines based on the Consolidated Health Economic Evaluation Reporting Standards (CHEERS) statement [17] were followed in this economic evaluation.

#### Study perspective

The analysis was conducted from an Australian public health provider perspective and included only costs borne by Australian Medicare and the South Australian Health Department.

#### Comparators

The intervention (enhanced general practice services) was compared to the control (usual care).

#### Time horizon

The analysis was based on a two-year time horizon with costs and outcomes between the two groups compared from the time of randomisation to the last follow-up (24 months).

#### Discount rates

In line with Australian guidelines [18], costs and outcomes were discounted at 5% [19].

#### Choice of outcomes

The primary outcome for the economic evaluation was the number of quality-adjusted life-years (QALYs) gained over 24 months (calculated using the trapezium method) [19–21].

Derived from the original EuroQol 5 Dimensions 3 Levels (EQ-5D-3L) questionnaire [16, 22], the EQ-5D-5L consists of five rather than three levels of impairment in each domain: no, slight, moderate, severe and extreme problems in the relevant dimension of health. Up to 3,125, different health states can be described using responses to the instrument. Utility values, ranging from -0.676 to 1, were estimated using an Australian value set [23]. A utility score equal to 1 represents ‘full health’ states, while one less than 0 represents health states that are worse than death [24].

#### Measurement of effectiveness

All effectiveness data analysed in this economic evaluation were obtained from the Flinders QUEST trial.

#### Estimating resources and cost

Resource use and costs were estimated from the Australian public health provider perspective. Out-of-hospital Medicare resource use and costs were estimated from Medical Benefits Schedule (MBS) data (to calculate costs of primary care visits, medical consultations, treatments, investigations, and allied health care visits). The cost of pharmaceuticals was estimated using Pharmaceutical Benefits Scheme (PBS) data. Costs of admission to public hospitals were estimated using Australian refined diagnosis-related groups (AR-DRG) data.

The cost of the intervention was set at $1,000 per participant. This was the quantum of payment received by practices for providing the enhanced general practice services to trial participants. All costs are reported in Australian dollars at 2021/22 unit prices.

#### Analytical methods

Analyses were conducted in MS Excel and Stata version 17.0 [25].

#### Base case analysis

The primary outcome was calculated using responses to the EQ-5D-5L questionnaire. As individuals did not complete EQ-5D-5L questionnaires at 24 months, 12-month responses were extrapolated to 24 months under the assumption that the effectiveness of the intervention gains at 12 months was sustained at 24 months. Therefore, 12-month EQ-5D-5L scores were extrapolated to the 24-month time point for each surviving patient in the intervention group. Participants in the Child cohort (who completed the proxy or youth versions of the EQ-5D questionnaire) were not included in the analysis. Best practice economic evaluation was conducted to establish whether the intervention was value for money when compared to usual care. An incremental approach was used to determine, where appropriate, the incremental cost-effectiveness ratios (ICERs) expressed as the incremental cost per QALY gained. The ICERs were calculated by dividing the difference in total costs (incremental costs) by the difference in the QALY gains (incremental effect) [26]. An intention-to-treat approach [18] was taken in the analysis.

The statistical analysis was based on a linear mixed model (LMM) and carried out under the assumption that any missing data were missing at random [27]. Inferences based on the LMM are valid when data are assumed to be MAR. Analyses comparing QALY gains over the trial period also controlled for baseline differences in EQ-5D-5L utility [25] within the LMM by including the baseline EQ5D-5L score as a covariate.

Within-trial economic evaluation with respect to QALYs was conducted, allowing for bivariate uncertainty with bootstrapping of participant costs and effects to maintain the covariance structure. Mean (standard error) and mean differences in costs and outcomes are reported with 95% bootstrapped confidence intervals (95% CI). Non-parametric bootstrapping [28, 29] was used to determine 1,000 paired estimates of mean differences in costs and outcomes from participant-level data. These bootstrapped pairs are presented as cost-effectiveness planes (CEPs) [30].

To further characterise the uncertainty in the economic evaluation results, cost-effectiveness acceptability curves (CEACs) were constructed. These CEACs show the probability of the intervention arm being cost-effective compared to the control arm at different willingness-to-pay (WTP) thresholds. A WTP threshold of $50,000 per QALY gained was used. This is the implicit criterion used for assessing the cost-effectiveness of new pharmaceuticals and medical services in Australia [14].

#### Sensitivity analyses

Two sensitivity analyses examining the impact of varying the intervention effectiveness assumptions were conducted. The first examined the impact of assuming a 5% drop in the effectiveness of the intervention from 12 to 24-month follow-up. The second considered a slightly bigger drop in effectiveness of 15%. For post-intervention periods, costs comprised just those for DRG, PBS and MBS resource use. Post-baseline 2-year costs and QALYs were discounted at 5% as per Australian recommendations [18].

#### Subgroup analysis

Subgroup analyses were performed for the adults (participants aged between 18–64 years with two or more chronic illnesses: *n* = 315) and the older adults (participants aged 65 years and over: *n* = 671) cohorts.

## Results

### Hospital service use

Descriptive statistics and estimated intervention effects for hospital service use are presented in Table 2. Over the two-year period starting from the commencement of the intervention (i.e. the 24-month follow-up period), there were no statistically significant differences between the control and intervention groups in the incidence of emergency department presentations (IRR 0.90; 95% confidence interval [CI] 0.71 to 1.15; *p* = 0.41), hospital admissions (IRR 0.95; 95% CI 0.72 to 1.27; *p* = 0.75) or the number of nights in hospital (IRR 0.80; 95% CI 0.45 to 1.43; *p* = 0.46).

**Table 2:** Hospital service use for the entire cohort

	**Control**			**Intervention**			**Intervention effect**^**a**^	
	**Baseline**	**12 months**	**24 months**	**Baseline**	**12 months**	**24 months**	**IRR (95% CI)**	***P*-value**
**No. patients in the analysis**	509	509	509	519	519	519		
**No. ED presentations**	341	361	312	364	343	297	0.90 (0.69 to 1.17)^b^	0.43
Mean (SD)	0.67 (1.44)	0.71 (1.57)	0.61 (1.26)	0.70 (1.72)	0.66 (1.64)	0.57 (1.46)	0.90 (0.71 to 1.15)^c^	0.41
**No. admissions**	239	277	228	228	241	228	0.90 (0.66 to 1.22)^b^	0.49
Mean (SD)	0.47 (1.04)	0.54 (1.21)	0.45 (1.02)	0.44 (1.01)	0.46 (1.08)	0.44 (1.15)	0.95 (0.72 to 1.27)^c^	0.75
**Night stays**	880	910	576	661	772	631	0.65 (0.34 to 1.24)^b^	0.19
Mean (SD)	1.73 (7.70)	1.79 (5.97)	1.13 (4.07)	1.27 (4.97)	1.49 (8.55)	1.22 (4.91)	0.80 (0.45 to 1.43)^c^	0.46

Data are the number and mean (standard deviation [SD]) per patient of hospital emergency department (ED) presentations, admissions and night stays

^a^The intervention effect (incidence rate ratio [IRR]) is calculated from a multilevel negative binomial regression model for the difference between the control and intervention groups over ^b^one or ^c^two years. The dataset comprises 1028 (98.5%) of 1044 patients who were matched to SA Health hospital records. Baseline, the 12-month period prior to the intervention; 12 months, the 12-month intervention period; 24 months, the 12-month period following the intervention period; CI, confidence interval

Subgroup analyses of hospital service use (Supplementary Table 1, Additional file 3) at the 24-month follow-up in the adults and older adults cohorts separately did not find any statistically significant intervention effects; the child cohort was too small to estimate an effect reliably. For the older adults cohort, there were non-significant intervention effect decreases of 21% for the incidence of emergency department presentations (IRR 0.79; 95% C, 0.58 to 1.06; *p* = 0.11), 22% for hospital admissions (IRR 0.78; 95% CI 0.55 to 1.09; *p* = 0.15) and 31% for the number of nights in hospital (IRR 0.69; 95% CI 0.34 to 1.31; *p* = 0.24).

### Specialist services

Descriptive statistics and estimated intervention effects for the number of specialist services are presented in Table 3. Over the two-year period starting from the commencement of the intervention (i.e. the 24-month follow-up period), there were no statistically significant differences between the control and intervention groups for the number of specialist services (IRR 0.99; 95% CI 0.91 to 1.09; *p* = 0.90).

**Table 3:** Specialist services and pharmaceuticals for the entire cohort

	**Control**			**Intervention**			**Intervention effect**^**a**^	
	**Baseline**	**12 months**	**24 months**	**Baseline**	**12 months**	**24 months**	**IRR (95% CI)**	***P*-value**
**No. patients in the analysis**	501	501	501	514	514	514		
**Specialist services**	18,205	17,729	16,336	18,904	18,349	17,179	0.99 (0.89 to 1.09)^b^	0.78
Mean (SD)	36.34 (37.23)	35.39 (49.04)	32.61 (35.82)	36.78 (31.79)	35.70 (35.59)	33.42 (41.44)	0.99 (0.91 to 1.09)^c^	0.90
**Pharmaceuticals**	26,790	26,780	26,613	27,449	27,294	27,124	1.00 (0.94 to 1.06)^b^	0.78
Mean (SD)	53.47 (37.44)	53.45 37.32	53.12 39.71	53.40 37.24	53.10 36.93	52.77 38.20	1.00 (0.95 to 1.04)^c^	0.94

Data are the number and mean (standard deviation [SD]) per patient of specialist services and pharmaceutical items supplied

^a^The intervention effect (incidence rate ratio [IRR]) is calculated from a multilevel negative binomial regression model for the difference between the control and intervention groups over ^b^one or ^c^two years. The dataset comprises 1015 (97.2%) of 1044 patients who were matched to Services Australia records. Baseline, the 12-month period prior to the intervention; 12 months, the 12-month intervention period; 24 months, the 12-month period following the intervention period; CI, confidence interval

Subgroup analyses of specialist services (Supplementary Table 2, Additional file 3) at the 24-month follow-up did not find any statistically significant differences between the control and intervention groups for the total number of specialist services in the child cohort (IRR 0.76; 95% CI 0.44 to 1.29; *p* = 0.30), adult cohort (IRR 1.00; 95% CI 0.86 to 1.17; *p* = 0.99) or older adult cohort (IRR 0.99; 95% CI 0.90 to 1.11; *p* = 0.97).

### Pharmaceuticals

Descriptive statistics and estimated intervention effects for the number of pharmaceuticals are presented in Table 3. Over the two-year period starting from the commencement of the intervention (i.e. the 24-month follow-up period), there were no statistically significant intervention effects for the number of pharmaceuticals (IRR 1.00; 95% CI 0.95 to 1.04; *p* = 0.94).

Subgroup analyses of the number of pharmaceuticals are presented in Supplementary Table 3, Additional file 3. At the 24-month follow-up in the child cohort, the intervention was associated with a statistically significant reduction (47%) in the number of pharmaceuticals (IRR 0.53; 95% CI 0.28 to 0.99; *p* = 0.045). This intervention effect occurred through an increase in the number of pharmaceuticals in the control group, whereas pharmaceuticals in the intervention group remained relatively constant. At the 24-month follow-up, there were no statistically significant differences between the control and intervention groups for the number of pharmaceuticals in either the adult (IRR 1.02; 95% CI 0.92 to 1.13; *p* = 0.68) or older adult (IRR 1.00; 95% CI 0.96 to 1.05; *p* = 0.96) cohorts.

### Economic evaluation

**Fig. 1:**
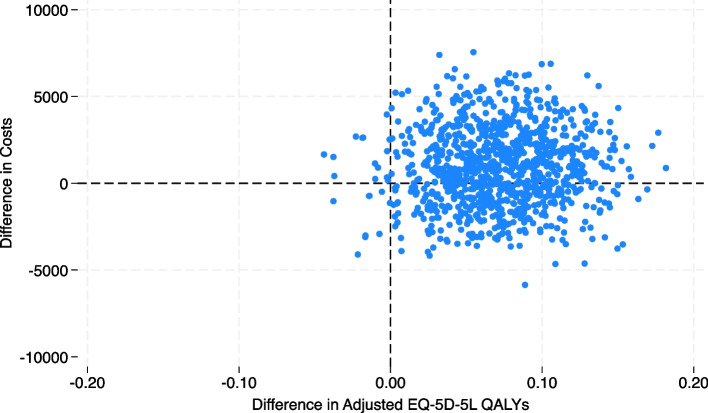
Cost-effectiveness plane (quality-adjusted life years [QALYs] gained over 24 months) for the adults and older adults cohorts combined. The cost-effectiveness plane shows the relationship between the incremental cost and incremental outcomes (QALYs gained at 24 months) of the intervention compared with control. It shows considerable uncertainty in the results because they are spread in all four quadrants. EQ-5D-5L, EuroQoL 5 dimensions, 5 levels questionnaire

**Fig. 2:**
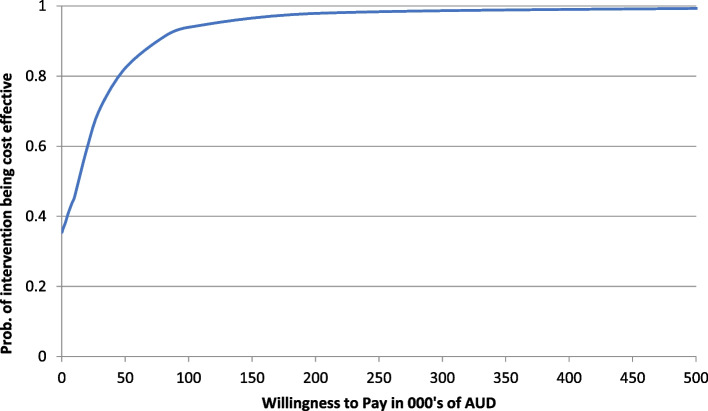
Cost-effectiveness acceptability curve (quality-adjusted life years [QALYs] gained over 24 months) for the adults and older adults cohorts combined. The figure shows the cost-effectiveness acceptability curve of the intervention versus usual care over 24 months when intervention costs are not included in the analysis. It shows that the probability of the intervention being cost-effective compared with usual care was approximately 80% if decision makers were willing to pay at $50,000 per QALY gained

The economic evaluation compared the relative cost-effectiveness of the intervention to usual care in the adult and older adult cohorts combined. At the 24-month follow-up (Table 4), the mean total cost per patient (including the intervention payment) was greater for the intervention than the control group by A$1237 (95% CI –A$4249 to A$6722; *p* = 0.659). However, the number of QALYs gained was higher for the intervention group (0.073; 95% CI –0.007 to 0.153; *p* = 0.072), and the estimated ICER was A$16,851 (95% CI A$11,468 to A$22,234) per QALY gained. This is lower than the A$50,000 per QALY gained threshold for determining cost-effectiveness. Therefore, the intervention at the 24-month follow-up was considered cost-effective. In the CEP (Fig. 1), bootstrapped cost and QALY difference estimates were distributed across all four quadrants, with a predominant concentration in the northeast quadrant. This finding implies that the intervention was associated with both increased costs and enhanced effectiveness. A CEAC (Fig. 2) showed that the probability of the intervention being cost-effective compared with usual care at a WTP threshold of A$50,000 per QALY gained was just over 80%.

**Table 4:** Mean costs and EQ-5D-5L outcomes per patient in the adults and older adults cohorts combined

	**Control**		**Intervention**		**Intervention effect**^**A**^	
	**Baseline**	**24 months**	**Baseline**	**24 months**	**Coefficient (95% CI)**	***P*-value**
**Costs (A$)**						
Hospital	5264 (583)	7554 (1120)	3887 (1041)	5963 (1023)	–214 (–4743 to 4316)	0.926
Specialist services	3203 (119)	6193 (123)	3378 (57)	6558 (141)	189 (–115 to 492)	0.222
Pharmaceuticals	2273 (119)	5211 (237)	2272 (387)	5305 (639)	95 (–1334 to 1524)	0.896
Intervention	0	0	0	1,026	1026 (1026 to 1026)	< 0.001
Total costs	10,792 (360)	18,007 (763)	9094 (1362)	17,546 (1524)	1237 (–4249 to 6722)	0.659
**Outcome**^**a**^						
EQ-5D-5L	0.607 (0.015)	0.584 (0.015)	0.635 (0.014)	0.620 (0.014)		
QALYs gained		1.150 (0.038)		1.218 (0.028)	0.068 (–0.014 to 0.150)	0.104

Data are mean (standard error [SE]) costs in Australian dollars (A$)

^A^The intervention effect is calculated from a multilevel linear regression model. Quality-adjusted life years (QALYs) were calculated from responses on the EuroQoL 5 dimensions, 5 levels (EQ-5D-5L) and questionnaire were adjusted for baseline differences. Baseline, the 12-month period prior to the intervention; 24 months, the 2-year period beginning from the start of the intervention

^a^In the base case, an assumption was made that 12 month EQ5D-5L scores were sustained at 24 months. In the QALY calculation, EQ5S-5L values at 24 months were adjusted for the number of decedents (who were assigned values of 0). QALYs were also discounted at 5%

Two sensitivity analyses examined the impact of varying the intervention effectiveness assumption while costs remained unchanged. If costs remained unchanged and the incremental effectiveness of the intervention declined by 5% at the 24-month time point (i.e. if the extrapolated 24-month EQ-5D-5L score for each surviving patient in the intervention group was 95% of the 12-month EQ-5D-5L value), the intervention would be associated with an ICER of A$21,031 per patient (95% CI A$13,126 to A$28,937), which is still lower than the WTP threshold of A$50,000 per QALY gained. If costs remained unchanged and the incremental effectiveness of the intervention declined by 15% at the 24-month time point (i.e. if the extrapolated 24-month EQ-5D-5L score for each surviving patient in the intervention group was 85% of the 12-month EQ-5D-5L value), the intervention would be associated with an ICER of A$41,743 per patient (95% CI -A$473,540 to A$557,026), which is still lower than the WTP threshold of A$50,000 per QALY gained.

Subgroup analyses of the economic evaluation are presented in Supplementary Table 5, Additional file 4.

In the adults cohort, the intervention was more expensive than the control, by A$5458 (95% CI –A$6101 to A$17,017; *p* = 0.355) per patient. The intervention was more effective than usual care in terms of EQ-5D-5L-based QALYs because it was associated with 0.036 (95% CI –0.020 to 0.092; *p* = 0.213) more QALYs gained per patient. The resulting ICER was estimated at A$152,820 per QALY gained (95% CI A$145,379 to A$160,262), which is higher than the A$50,000 per QALY gained threshold for determining cost-effectiveness used in Australia [14]. In the CEP (Supplementary Fig. 1, Additional file 4) bootstrapped cost and QALY difference estimates were distributed across all four quadrants indicating considerable uncertainty in the results. A CEAC (Supplementary Fig. 2, Additional file 4) showed that at 24 months the probability of the intervention being cost-effective compared with usual care at a WTP threshold of A$50,000 per QALY gained was only approximately 46%.

For the older adults cohort, the intervention was cheaper than usual care by A$540 (95% CI –A$3412 to A$2331; *p* = 0.712) per patient. The savings occurred primarily through lower hospital costs (–A$1070; 95% CI –A$3732 to A$1592; *p* = 0.431). The intervention was again more effective than usual care in terms of EQ-5D-5L-based QALYs because it was associated with 0.080 (95% CI –0.030 to 0.190; *p* = 0.156) more QALYs gained per patient. Therefore, the intervention was both cheaper and more effective than usual care. In the CEP (Supplementary Fig. 3, Additional file 4) bootstrapped cost and QALY difference estimates were distributed across all four quadrants indicating considerable uncertainty in the results. A CEAC (Supplementary Fig. 4, Additional file 4) showed that the probability of the intervention being cost-effective compared with usual care at a WTP threshold of A$50,000 per QALY gained was approximately 95%.

## Discussion

The results from the two-year follow-up of Flinders QUEST are broadly consistent with those observed at 12 months [13]. At both the 12- and 24-month follow-ups, we failed to detect statistically significant differences between the control and intervention groups with respect to our health service outcomes. At the 12-month follow-up, cost-effectiveness was shown for the older adults (≥ 65 years) cohort (*n* = 671). With the longer follow-up period of two years, we found that the intervention had a more than even chance (about 80%) of being cost-effective for the total sample at the $50,000/QALY cost-effectiveness threshold. For the older cohort, the intervention dominated usual care with both lower costs (due to lower hospital costs) and increased effectiveness, as measured by QALYs.

Discordant results between the clinical and economic evaluations are not uncommon in clinical trials that include economic evaluations [31]. In the present study the discordant results lead to the seemingly paradoxical conclusion that while the intervention was not clinically effective it was probably cost-effective. The discrepancy between the two sets of results arises because economic evaluations focus on the likelihood of an intervention being cost‐effective, in contrast to the null hypothesis testing used to evaluate clinical interventions [17, 28, 29, 31, 32]. Cost‐effectiveness analyses evaluate costs and effects together and decisions are based on the likelihood of cost-effectiveness irrespective of whether differences are statistically significant [32].

### Strengths and limitations of the study

The key strengths of Flinders QUEST were its cluster randomised controlled trial study design, the high standard of implementation and the high-quality data. The major limitations related to the relatively short intervention period (12 months), the fact that the practices were drawn from an academic practice research network, which may not be representative of Australian practices [33], and that the trial was not adequately powered to detect changes in hospital use.

The longer 24-month follow-up period partially addressed the issue of the lack of power to detect changes in hospital use by extending the length of the follow-up period from 12 to 24 months. Despite this while the estimates of the intervention effect across the hospital use measures, particularly for the older cohort, were generally of an order of magnitude that would be considered meaningful in the Australian context [34] they were not statistically significant. The very wide 95% CIs indicate that the estimates are imprecise and that a larger sample size would be required [35].

A key limitation of the 24-month follow-up study is that patient self-report questionnaire data were not collected at 24 months. This meant that the primary outcome for the study (patient self-rated health status, measured by the VAS of the EQ-5D questionnaire) and the primary outcome for the cost-effectiveness analysis (the number of QALYs) could not be updated for the 24-month analysis. The reason that 24-month follow-up data was not collected from patients was primarily due to funding constraints, specifically, the very high staff cost involved in collecting data from a significant number of older patients via face-to-face home interviews.

In our main economic analysis, it was assumed that the effectiveness shown at 12 months was sustained through to 24 months. Two sensitivity analyses tested the impact of varying this intervention effectiveness assumption: a 5% and a 15% drop in the effectiveness of the intervention at the 24-month follow-up. Both sensitivity analyses produced substantively the same result as the main analysis. Importantly, in the older adults cohort, even if it were assumed that there was no difference between the control and intervention groups with respect to QALYs gained at 24 months, the intervention would still have been cost-effective because the intervention was associated with lower healthcare costs (including the A$1000 cost of the intervention) of –A$540 per patient.

### Impact of COVID-19

The first recorded South Australian case of COVID-19 was reported on 1 February 2020, so the pandemic did not affect the study during the intervention period (1 November 2018 – 31 October 2019). From March 2020 South Australia experienced state border closures and restrictions regarding the size of gatherings. Between March 2020 to October 2020 (during the 24-month follow-up) it is possible that COVID-19 impacted on the 24-month health service utilisation outcomes reported in the present study although we have no evidence that COVID-19 differentially affected patients in either the control or intervention groups.

Consistent with the experiences reported in another Australian State [36] and internationally in the UK [37] and the US [38, 39] there appeared to be an overall reduction in the number of ED presentations amongst our participants in both the control and intervention arms during the initial stages of the pandemic. The number of Medicare specialist appointments and pharmaceutical items supplied on the other hand showed little change during the 24-month follow-up period compared to the 12-month intervention period and baseline (the 12 month period prior to the intervention). Overall, our results support the view that a proportion of ED presentations may have a discretionary element [40, 41] and that the pandemic has illustrated that reductions in ED presentations may be feasible through alternative models of care such as a greater use of telehealth in primary care [42].

## Conclusions

The results from the two-year follow-up of patients in Flinders QUEST continue to be mixed and the benefit of adding the intervention to usual general practice care for patients at high risk of poor health outcomes remains unclear. However, in the older adults cohort, the intervention was found to be likely cost-effective at 12 months, and this finding strengthened in the 24-month follow-up. We believe that the positive cost-effectiveness results from the 24-month follow-up warrant replication in further studies. These studies should be appropriately powered for health service use, with an intervention period of at least two years, and targeted to older people at high risk of poor health outcomes.

### Supplementary Information

**Supplementary Material 1. **

**Supplementary Material 2. **

**Supplementary Material 3. **

**Supplementary Material 4. **

## Data Availability

The datasets generated and analysed during the present study are not publicly available due to the potential for identifying participants. The datasets are available from the corresponding author upon reasonable request.
