# Supplementary material for: Two-year follow-up of a clustered randomised controlled trial of a multicomponent general practice intervention for people at risk of poor health outcomes
Source: BMC Health Serv Res. 2024 Apr 19;24:488. doi: 10.1186/s12913-024-10799-2 (PMC11031969; doi:10.1186/s12913-024-10799-2)
Supplement: Supplementary file 1 — Supplementary Material 1. [file 12913_2024_10799_MOESM1_ESM.docx]

**Additional File 1: Methodological background to Flinders QUEST**

Full methodological details to Flinders QUEST can be found in the main paper of the 12-month outcome results [1]. Here we briefly summarise several of the key aspects of the trial reported in this earlier paper to aid interpretation of the two-year follow-up results.

Flinders QUEST was a parallel group cluster randomised controlled trial (RCT). General practices located in the metropolitan area of Adelaide, South Australia were randomised to control (usual care) or intervention (enhanced care) groups. The cluster design was chosen to ensure that all patients within a general practice were assigned to the same trial arm, thereby reducing the risk of contamination of the intervention effect.

The general practices were recruited from the Flinders General Practice Teaching and Research Network which at the time of the study comprised 53 general practices. To be eligible for the study GPs were required to work at least three days per week in the practice. A representative from each practice signed a Memorandum of Understanding to take part in the study.

Participants were active patients at the general practices. Practices used the Pen CS Clinical Auditing Tool (CAT4) to produce lists of potentially eligible participants drawn from one of three cohorts - children and young people (under 18 years), adults (18–64 years) with two or more chronic diseases, and older people (65 years or more). GPs identified potentially eligible patients as those at high risk of poor health outcomes and invited them to express an interest in taking part in the study by contacting the researchers. The key patient exclusion criteria were a disease likely to shorten life expectancy, non-English speaking, cognitive impairment, severe mental illness and not community dwelling. Participants provided written consent prior to randomisation.

The multicomponent intervention comprised enrolment of patients with a preferred GP, longer general practice appointments, and general practice follow-up within seven days of emergency department and hospital care episodes. Intervention practices received payment of $1000 per enrolled participant.

The primary outcome of Flinders QUEST was change in self-rated health (measured by the VAS of the EQ-5D) between baseline and 12-month follow-up for control (usual care) and intervention groups. Secondary outcomes included the numbers of emergency department presentations and hospital admissions, Medicare specialist claims and Pharmaceutical Benefits Scheme (PBS) items supplied, Health Literacy Questionnaire scores, and cost-effectiveness of the intervention (based on the number of quality-adjusted life-years [QALYs] gained over 12 months, derived from EQ-5D utility scores.

The power calculation was based on detecting a standardised effect size on the VAS of between 0.3 and 0.5 standard deviation. Assuming 10 general practices per arm with a total of 1000 patients power was estimated at 92% to detect a VAS 0.3-standard deviation change for the total (all cohorts) sample. Practices were randomised (by a statistician independent of the research team) using a covariate-constrained technique which included covariates for the size of practice and the Socio-Economic Indexes for Areas Index of Relative Socio-Economic Disadvantage (IRSD) score based on the practice postcode. Due to the design of study, there was no blinding to group allocation.

Twenty practices with a total of 92 GPs were recruited between 23 January 2018 –19 February 2018. No practices withdrew during the study. GPs invited 1656 patients to take part in the study of whom 1044 consented between 30 April 2018 –10 October 2018. At the 12-month follow-up patient level data were available for 965 patients with attrition being mainly due to the patient becoming uncontactable (26), death (22) or too ill to continue in the study (18).

At baseline the general practices ranged in size from one to ten full-time equivalent GPs and privately owned. All practices employed at least one practice nurse. Practices were drawn from areas of low, middle and high socio-economic status. Of the 92 GPs enrolled at baseline most (76) had graduated in Australia, were men (50) and on average had 17.7 years (SD, 11.3) of general practice experience. At baseline the mean age of the 1044 patients was 64.6 (SD, 19.3) years, and 562 were girls or women (54%). Most (671) patients were recruited from the older (≥65 years) cohort; only a small number (58) of children or young people were recruited. Of the 986 adults and older adults, 595 were married or in a de facto relationship, and 613 were retired. Patients reported a mean of 3.4 (SD, 1.5) chronic diseases, the most frequent types being cardiovascular (627) and musculoskeletal disorders (616).

A process evaluation was conducted alongside the trial to assess the quality of the intervention delivered by intervention group practices. The results from the process evaluation indicated that the intervention was associated with modest but statistically significant improvements to the likelihoods of GP appointments with the most frequently seen GP (continuity of care) and longer length appointments but not of timely follow-up after emergency department or hospital care episodes.

1. Reed RL, Roeger L, Kwok YH, Kaambwa B, Allison S, Osborne RH. A general practice intervention for people at risk of poor health outcomes: the Flinders QUEST cluster randomised controlled trial and economic evaluation. Med J Aust. 2022;216(9):469–475. [10.5694/mja2.51484](https://doi.org/10.5694/mja2.51484).
