# Supplementary material for: Two-year follow-up of a clustered randomised controlled trial of a multicomponent general practice intervention for people at risk of poor health outcomes
Source: BMC Health Serv Res. 2024 Apr 19;24:488. doi: 10.1186/s12913-024-10799-2 (PMC11031969; doi:10.1186/s12913-024-10799-2)
Supplement: Supplementary file 2 — Supplementary Material 2. [file 12913_2024_10799_MOESM2_ESM.docx]

**Additional File 2: Hospital service use, specialist services and pharmaceuticals: data sources and methods**

*Hospital service use*

Hospital service use was derived from SA Health public hospital administrative records held in the SA Health Emergency Department Data Collection (EDDC) and the SA Health Integrated South Australian Activity Collection (ISAAC). Private hospitals are not covered in these databases.

Of the 1044 Flinders QUEST patients, 1028 (98.5%) could be correctly matched to Patient Master Index entries using a combination of date of birth and first and last name, with address used in a second stage of further checking. Match rates were similar between the control (98.3%) and intervention (98.7%) groups, and the difference in group match rates was not statistically significant (χ^2^ = 0.29; df = 1; *p* = 0.59).

Multilevel negative binomial regression was used to analyse the counts of the hospital service use indicators. The multilevel models were estimated with Stata v17.0 using the mixed model suite of commands. The Stata command for the hospital utilisation analyses (using emergency department [ED] presentations by way of example) took the form: “menbreg emergency i.time##i.group, exposure(expose) ||practice: ||id:, irr”. Note that random effects to account for clustering at the practice level and the repeated measurement (the two time periods) at the patient level were included in the models.

The models included an exposure variable to account for patients having different lengths of follow-up due to death. At 24 months (31 October 2020), for the hospital service dataset (*n* = 1028), there were 37 deaths in total (control = 16; intervention = 21). Mean exposure times were similar between the control and intervention groups (mean 710 and 712 days, respectively).

The intervention effect (incidence rate ratio [IRR]) in the models is shown by the non-linear interaction term between time (coded as 0 for the 12-month period before the intervention and as 1 for the comparison period) and group (coded as 0 for the control group and as 1 for the intervention group) [1].

Two intervention effects are reported in the results. The first intervention effect reported is the difference between the control and intervention groups over the 12-month intervention period controlling for baseline (the 12-month period prior to the intervention). The second intervention effect reported is the difference between the control and intervention groups over 24 months (which encompasses the 12-month intervention period and the 12-month period following the intervention period), controlling for baseline (the 12-month period prior to the intervention).

The intervention effect (in percentage terms) was defined as 1 – IRR × 100. For example, an IRR of 1.25 represents an increase of 25%; an IRR of 0.85 represents a decrease of 15%.

*Specialist services and pharmaceuticals*

In Australia, the Australian Government funds a national universal health insurance scheme known as Medicare. Medicare services are provided to all Australian residents who hold a current Medicare card and overseas visitors from countries with which Australia has a reciprocal health care agreement. Medicare provides treatment in public hospitals free of charge and subsidises out-of-hospital treatment through a rebate benefit. The Pharmaceutical Benefits Scheme (PBS) is similarly funded by the Australian Government to provide subsidised pharmaceuticals that have been listed on the PBS schedule. Medicare and PBS data are regarded as very accurate because healthcare providers and patients are strongly motivated to provide data to receive payments.

Medicare and PBS data were sourced from Services Australia. Of the 1044 patients in the trial, Medicare and PBS datasets were created comprising 1015 (97.2%) patients. The most frequent reason for patients not being included in the analysis datasets (*n* = 29) was that the patient held a Department of Veterans Affairs card and did not have any Medicare or PBS records. The patient exclusions from the Medicare and PBS analysis datasets did not differ substantively between groups, with 96.7% and 97.7% of patients in the control and intervention groups, respectively, matched. The difference in matching rates was not statistically significant (χ^2^ = 0.97; df = 1; *p* = 0.33).

Medicare specialist services for the purposes of the study were defined as all Medicare claims other than those claimed by general practitioners (GPs). This included claims categorised as diagnostic, therapeutic, surgical, imaging, pathology, optometry, dental, psychology/psychiatry, specialists and allied health. For Medicare claims, the date on which the analysis was based was the date the service was provided; for the PBS, it was the date the PBS item was supplied.

The statistical technique used for the analysis of Medicare claims and PBS items supplied followed that used in the hospital service use analyses (see above) and was based on multilevel negative binomial regression. The models included an exposure variable to account for patients having different durations of follow-up due to death. At 24 months (31 October 2020), for the Medicare and PBS datasets (*n* = 1015), there were 32 deaths in total (control = 14; intervention = 18). Mean exposure times were similar between the control and intervention groups (mean 712 and 716 days, respectively).

**References**

1. Puhani PA. The treatment effect, the cross difference, and the interaction term in nonlinear “difference-in-differences” models. Econ Lett. 2012;115(1):85–87.
