# Supplementary material for: Two-year follow-up of a clustered randomised controlled trial of a multicomponent general practice intervention for people at risk of poor health outcomes
Source: BMC Health Serv Res. 2024 Apr 19;24:488. doi: 10.1186/s12913-024-10799-2 (PMC11031969; doi:10.1186/s12913-024-10799-2)
Supplement: Supplementary file 3 — Supplementary Material 3. [file 12913_2024_10799_MOESM3_ESM.docx]

**Additional File 3: Sub-group analyses**

**Supplementary Table 1** **Hospital service use by cohort**

|  | **Control** | | | **Intervention** | | | **Intervention effect^A^** | |
| --- | --- | --- | --- | --- | --- | --- | --- | --- |
|  | **Baseline** | **12 months** | **24 months** | **Baseline** | **12 months** | **24 months** | **IRR (95% CI)** | ***P*-value** |
| **Child cohort** |  |  |  |  |  |  |  |  |
| No. patients in the analysis | 27 | 27 | 27 | 30 | 30 | 30 |  |  |
| ED presentations | 35 | 25 | 20 | 33 | 28 | 22 |  |  |
| Mean (SD) | 1.30 (2.76) | 0.93 (1.59) | 0.74 (1.16) | 1.10 (1.81) | 0.93 (1.70) | 0.73 (1.01) |  |  |
| Admissions | 16 | 10 | 8 | 16 | 13 | 8 |  |  |
| Mean (SD) | 0.59 (1.31) | 0.37 (0.74) | 0.30 (0.82) | 0.53 (1.07) | 0.43 (1.19) | 0.27 (0.64) |  |  |
| Nights stay | 81 | 12 | 3 | 27 | 37 | 13 |  |  |
| Mean (SD) | 3.00 (13.66) | 0.44 (1.45) | 0.11 (0.42) | 0.90 (2.96) | 1.23 (5.13) | 0.43 (1.52) |  |  |
| **Adults cohort** |  |  |  |  |  |  |  |  |
| No. patients in the analysis | 169 | 169 | 169 | 141 | 141 | 141 |  |  |
| ED presentations | 124 | 115 | 82 | 110 | 108 | 95 | 1.05 (0.63 to 1.76)^B^ | 0.85 |
| Mean (SD) | 0.73 (1.51) | 0.68 (1.68) | 0.49 (1.02) | 0.78 (1.95) | 0.77 (2.29) | 0.67 (2.20) | 1.06 (0.66 to 1.71)^C^ | 0.81 |
| Admissions | 83 | 77 | 48 | 52 | 60 | 62 | 1.25 (0.68 to 2.31)^B^ | 0.48 |
| Mean (SD) | 0.49 (1.13) | 0.46 (1.27) | 0.28 (0.91) | 0.37 (0.81) | 0.43 (1.12) | 0.44 (1.48) | 1.52 (0.87 to 2.65)^C^ | 0.14 |
| Nights stay | 278 | 285 | 175 | 141 | 288 | 207 | 0.82 (0.20 to 3.38)^B^ | 0.79 |
| Mean (SD) | 1.64 (9.03) | 1.69 (7.47) | 1.04 (4.40) | 1.00 (3.52) | 2.04 (14.78) | 1.47 (6.02) | 1.00 (0.28 to 3.64)^C^ | 0.99 |
| **Older adults cohort** |  |  |  |  |  |  |  |  |
| No. patients in the analysis | 313 | 313 | 313 | 348 | 348 | 348 |  |  |
| ED presentations | 182 | 221 | 210 | 221 | 207 | 180 | 0.80 (0.58 to 1.12)^B^ | 0.20 |
| Mean (SD) | 0.58 (1.21) | 0.71 (1.50) | 0.67 (1.37) | 0.64 (1.62) | 0.59 (1.27) | 0.52 (1.07) | 0.79 (0.58 to 1.06)^C^ | 0.11 |
| Admissions | 140 | 190 | 172 | 160 | 168 | 158 | 0.76 (0.52 to 1.11)^B^ | 0.16 |
| Mean (SD) | 0.45 (0.96) | 0.61 (1.21) | 0.55 (1.09) | 0.46 (1.07) | 0.48 (1.06) | 0.45 (1.02) | 0.78 (0.55 to 1.09)^C^ | 0.15 |
| Nights stay | 521 | 613 | 398 | 493 | 447 | 411 | 0.55 (0.26 to 1.15)^B^ | 0.11 |
| Mean (SD) | 1.66 (6.07) | 1.96 (5.26) | 1.27 (4.37) | 1.42 (5.58) | 1.28 (4.31) | 1.18 (4.59) | 0.69 (0.34 to 1.31)^C^ | 0.24 |

Data are the number and mean (standard deviation [SD]) per patient of hospital emergency department (ED) presentations, admissions and night stays. ^A^The intervention effect (incidence rate ratio [IRR]) is calculated from a multilevel negative binomial regression model for the difference between the control and intervention groups over ^B^one or ^C^two years. The dataset comprises *n* = 1028 (98.5%) of the 1044 patients who were matched to SA Health hospital records. Baseline, the 12-month period prior to the intervention; 12 months, the 12-month intervention period; 24 months, the 12-month period following the intervention period; CI, confidence interval.

**Supplementary Table 2** **Medicare specialist claims by cohort**

|  | **Control** | | | **Intervention** | | | **Intervention effect^A^** | |
| --- | --- | --- | --- | --- | --- | --- | --- | --- |
|  | **Baseline** | **12 months** | **24 months** | **Baseline** | **12 months** | **24 months** | **IRR (95% CI)** | ***P*-value** |
| **Child cohort** |  |  |  |  |  |  |  |  |
| No. patients in the analysis | 25 | 25 | 25 | 28 | 28 | 28 |  |  |
| Medicare specialist claims | 326 | 317 | 315 | 287 | 289 | 222 | 0.83 (0.45 to 1.52)^B^ | 0.54- |
| Mean (SD) | 13.04 (23.57) | 12.68 (14.07) | 12.60 (13.38) | 10.25 (10.77) | 10.32 (14.72) | 7.93 (10.69) | 0.76 (0.44 to 1.29)^C^ | 0.30 |
| **Adults cohort** |  |  |  |  |  |  |  |  |
| No. patients in the analysis | 169 | 169 | 169 | 143 | 143 | 143 |  |  |
| Medicare specialist claims | 5084 | 4282 | 4269 | 4815 | 4282 | 4222 | 0.96 (0.80 to 1.14)^B^ | 0.62 |
| Mean (SD) | 30.08 (28.23) | 25.34 (19.81) | 25.26 (26.56) | 33.67 (31.94) | 29.94 (27.88) | 29.52 (25.52) | 1.00 (0.85 to 1.17)^C^ | 0.99 |
| **Older adults cohort** |  |  |  |  |  |  |  |  |
| No. patients in the analysis | 307 | 307 | 307 | 343 | 343 | 343 |  |  |
| Medicare specialist claims | 12,795 | 13,130 | 11,752 | 13,802 | 13,778 | 12,735 | 1.00 (0.88 to 1.13)^B^ | 0.98 |
| Mean (SD) | 41.68 (41.09) | 42.77 (59.54) | 38.28 (40.00) | 40.24 (31.78) | 40.17 (38.38) | 37.13 (47.15) | 0.99 (0.90 to 1.11)^C^ | 0.97 |

Data are the number and mean (standard deviation [SD]) per patient of Medicare specialist claims. ^A^The intervention effect (incidence rate ratio [IRR]) is calculated from a multilevel negative binomial regression model for the difference between the control and intervention groups over ^B^one or ^C^two years. The dataset comprises *n* = 1015 (97.2%) of 1044 patients who were matched to Services Australia records. Baseline, the 12-month period prior to the intervention; 12 months, the 12-month intervention period; 24 months, the 12-month period following the intervention period; CI, confidence interval.

**Supplementary Table 3** **Pharmaceutical Benefits Scheme items supplied by cohort**

|  | **Control** | | | **Intervention** | | | **Intervention effect^A^** | |
| --- | --- | --- | --- | --- | --- | --- | --- | --- |
|  | **Baseline** | **12 months** | **24 months** | **Baseline** | **12 months** | **24 months** | **IRR (95% CI)** | ***P*-value** |
| **Child cohort** |  |  |  |  |  |  |  |  |
| No. patients in the analysis | 25 | 25 | 25 | 28 | 28 | 28 |  |  |
| PBS items supplied | 89 | 152 | 187 | 222 | 231 | 242 | 0.54 (0.29 to 0.98)^B^ | 0.041 |
| Mean (SD) | 3.56 (3.61) | 6.08 (4.09) | 7.48 (10.48) | 7.93 (7.54) | 8.25 (9.69) | 8.64 (11.07) | 0.53 (0.28 to 0.99)^C^ | 0.045 |
| **Adults cohort** |  |  |  |  |  |  |  |  |
| No. patients in the analysis | 169 | 169 | 169 | 143 | 143 | 143 |  |  |
| PBS items supplied | 8139 | 8181 | 8091 | 7507 | 7434 | 7427 | 1.01 (0.90 to 1.14)^B^ | 0.28 |
| Mean (SD) | 48.16 (37.11) | 48.41 (38.28) | 47.88 (41.19) | 52.50 (38.65) | 51.99 (38.25) | 51.94 (38.72) | 1.02 (0.92 to 1.13)^C^ | 0.68 |
| **Older adults cohort** |  |  |  |  |  |  |  |  |
| No. patients in the analysis | 307 | 307 | 307 | 343 | 343 | 343 |  |  |
| PBS items supplied | 18,562 | 18,447 | 18,335 | 19,720 | 19,629 | 19,455 | 1.01 (0.95 to 1.07)^B^ | 0.85 |
| Mean (SD) | 60.46 (35.57) | 60.09 (35.05) | 59.72 (37.51) | 57.49 (35.63) | 57.23 (35.33) | 56.72 (37.16) | 1.00 (0.96 to 1.05)^C^ | 0.96 |

Data are the number and mean (standard deviation [SD]) per patient of Pharmaceutical Benefits Scheme (PBS) items supplied. ^A^The intervention effect (incidence rate ratio [IRR]) is calculated from a multilevel negative binomial regression model for the difference between the control and intervention groups over ^B^one or ^C^two years. The dataset comprises *n* = 1015 (97.2%) of 1044 patients who were matched to Services Australia records. Baseline, the 12-month period prior to the intervention; 12 months, the 12-month intervention period; 24 months, the 12-month period following the intervention period; CI, confidence interval.
