# Supplementary material for: Two-year follow-up of a clustered randomised controlled trial of a multicomponent general practice intervention for people at risk of poor health outcomes
Source: BMC Health Serv Res. 2024 Apr 19;24:488. doi: 10.1186/s12913-024-10799-2 (PMC11031969; doi:10.1186/s12913-024-10799-2)
Supplement: Supplementary file 4 — Supplementary Material 4. [file 12913_2024_10799_MOESM4_ESM.docx]

**Additional File 4: Economic evaluation: cost data and sub-group results**

**Cost data**

For the Medicare and PBS costs, data were available for all MBS claims and PBS items supplied. For public hospital ED presentations and admissions, there were instances of missing cost data (Supplementary Table 4). The primary reason this occurred was that the event occurred in a hospital that was not part of the Southern Adelaide or Central Adelaide Local Health Network, where data sharing agreements enabled this cost information to be readily extracted. The difference in the rates of complete cost data between the control (75.4%) and intervention (73.9%) groups at 24 months was not statistically significant (χ^2^ = 0.27; df = 1; *p* = 0.60).

**Supplementary Table 4** **Missing cost data in hospital records (adults and older adults cohorts combined)**

|  | **Control** | | **Intervention** | |
| --- | --- | --- | --- | --- |
|  | **Baseline** | **24 months** | **Baseline** | **24 months** |
| **No. patients in analysis** | 490 | 490 | 496 | 496 |
| **No. ED presentations** | 306 | 628 | 331 | 512 |
| No. (%) with cost data | 297 (97.1) | 574 (91.4) | 318 (96.1) | 301 (86.8) |
| **No. hospital admissions** | 223 | 487 | 212 | 448 |
| No. (%) with cost data | 223 (100) | 266 (75.4) | 209 (98.6) | 222 (73.9) |

**Supplementary Table 5 Mean costs and EQ-5D-5L outcomes per patient by cohort**

|  | **Control** | | **Intervention** | | **Intervention effect^A^** | |
| --- | --- | --- | --- | --- | --- | --- |
|  | **Baseline** | **24 months** | **Baseline** | **24 months** | **Coefficient (95% CI)** | ***P*-value** |
| **Adults cohort** |  |  |  |  |  |  |
| Costs (A$) |  |  |  |  |  |  |
| Hospital | 6223 (1309) | 8190 (1292) | 3319 (2351) | 7629 (3123) | 2343 (–6845 to 11,532) | 0.617 |
| Medicare | 2835 (211) | 4653 (199) | 3049 (296) | 5855 (128) | 989 (–25 to 2002) | 0.056 |
| PBS | 1903 (287) | 3930 (191) | 1931 (369) | 4509 (245) | 551 (–813 to 1915) | 0.428 |
| Intervention | 0 | 0 | 0 | 1026 | 1026 (1026 to 1026) | <0.001 |
| Total costs | 10,945 (1112) | 15,596 (836) | 8177 (3025) | 18,287 (3325) | 5458 (–6101 to 17,017) | 0.355 |
| Outcome^a^ |  |  |  |  |  |  |
| EQ-5D-5L | 0.531 (0.029) | 0.555 (0.026) | 0.529 (0.027) | 0.572 (0.026) |  |  |
| QALYs gained |  | 1.058 (0.047) |  | 1.094 (0.047) | 0.036 (–0.020 to 0.092) | 0.213 |
| **Older adults cohort** |  |  |  |  |  |  |
| Costs (A$) |  |  |  |  |  |  |
| Hospital | 4683 (817) | 6902 (539) | 4247 (591) | 5396 (844) | –1070 (–3732 to 1592) | 0.431 |
| Medicare | 3429 (128) | 7060 (301) | 3473 (175) | 6805 (240) | –298 (–1389 to 793) | 0.592 |
| PBS | 2478 (122) | 5901 (650) | 2413 (320) | 5645 (607) | –190 (–1315 to 934) | 0.740 |
| Intervention | 0 | 0 | 0 | 1,026 | 1026 (1026 to 1026) | <0.001 |
| Total costs | 10,526 (404) | 18,806 (1092) | 9428 (433) | 17,168 (725) | –540 (–3412 to 2331) | 0.712 |
| Outcome^a^ |  |  |  |  |  |  |
| EQ-5D-5L | 0.644 (0.017) | 0.598 (0.018) | 0.677 (0.015) | 0.639 (0.017) |  |  |
| QALYs gained |  | 1.182 (0.041) |  | 1.262 (0.027) | 0.080 (–0.030 to 0.190) | 0.156 |

Data are mean (standard error [SE]) costs in Australian dollars (A$). ^A^The intervention effect is calculated from a multilevel linear regression model. Quality-adjusted life years (QALYs) were calculated from EQ-5D-5L responses and were adjusted for baseline differences. Baseline, the 12-month period prior to the intervention; 24 months, the two-year period beginning from the start of the intervention; CI, confidence interval; EQ-5D-5L, EuroQoL 5 dimensions, 5 levels questionnaire; PBS, Pharmaceutical Benefits Scheme.

^a^In the base case, an assumption was made that 12-month EQ5D-5L scores were sustained at 24 months. In the QALY calculation, EQ5S-5L values at 24 months were adjusted for the number of decedents (who were assigned values of 0). QALYs were also discounted at 5%.

Supplementary Figure 1 Cost-effectiveness plane (quality-adjusted life years [QALYs] gained over 24 months) for the adults cohort

The cost-effectiveness plane shows the relationship between the incremental cost and incremental outcomes (QALYs gained at 24 months) of the intervention compared with control. It shows considerable uncertainty in the results because they are spread in all four quadrants. EQ-5D-5L, EuroQoL 5 dimensions, 5 levels questionnaire.

Supplementary Figure 2 Cost-effectiveness acceptability curve (quality-adjusted life years [QALYs] gained over 24 months) for the adults cohort

The figure shows the cost-effectiveness acceptability curve of the intervention versus usual care over 24 months when intervention costs are not included in the analysis. It shows that the probability of the intervention being cost-effective compared with usual care was approximately 46% if decision makers were willing to pay at $50,000 per QALY gained.

Supplementary Figure 3 Cost-effectiveness plane (quality-adjusted life years [QALYs] gained over 24 months) for the older adults cohort

The cost-effectiveness plane shows the relationship between the incremental cost and incremental outcomes (QALYs gained at 24 months) of the intervention compared with control. It shows considerable uncertainty in the results because they are spread in all four quadrants. EQ-5D-5L, EuroQoL 5 dimensions, 5 levels questionnaire.

Supplementary Figure 4 Cost-effectiveness acceptability curve (quality-adjusted life years [QALYs] gained over 24 months) for the older adults cohort

The figure shows the cost-effectiveness acceptability curve of the intervention versus usual care over 24 months. It shows that the probability of the intervention being cost-effective compared with usual care was approximately 95% if decision makers were willing to pay at $50,000 per QALY gained.

**References**
